# Enhancing the Photocatalytic Activity of TiO_2_ Nanoparticles with Cyclodextrin-Functionalized Graphene and Noble Metals for Organic Pollutant Degradation

**DOI:** 10.3390/molecules31081296

**Published:** 2026-04-16

**Authors:** Ibtisam M. N. Hamdan, Mohannad T. Aljarrah, Nathir A. F. Al-Rawashdeh

**Affiliations:** 1 Chemistry Department, Jordan University of Science & Technology, Irbid 22110, Jordan; 2 Department of Chemical Engineering, Jordan University of Science & Technology, Irbid 22110, Jordan; 3 College of Engineering and Technology, University of Doha for Science and Technology, Doha P.O. Box 24449, Qatar

**Keywords:** TiO_2_ photocatalyst, β-cyclodextrin, reduced graphene oxide, methylene blue, nobel metal, water treatment

## Abstract

Contamination of water resources by organic pollutants is a major environmental issue. Utilizing photocatalytic materials for the degradation of these pollutants presents a viable strategy for environmental clean-up. This study introduces the synthesis of an organic/inorganic hybrid photocatalyst of β-cyclodextrin (β-CD)/reduced graphene oxide (rGO) and titanium oxide (TiO_2_) nanoparticles. The nanocomposite was characterized by using FT-IR, XRD, SEM, and EDAX, and the photocatalytic activity was studied by measuring the photodegradation of methylene blue (MB) under simulated solar radiation. The synthesized nanocomposite showed excellent stability and performance, with up to 92% photodegradation of MB. To further enhance the photocatalytic activity, the synthesized nanocomposite underwent modification with Ag and Pt nanoparticles. Within 90 min, photodegradation rates of 100% and 97% for MB were attained with Pt and Ag nanoparticles that were loaded at 5 wt.%, respectively. The photocatalyst’s reusability was evaluated through multiple usage cycles. Additionally, the impact of functionalization on the band gap alteration of TiO_2_ is reported.

## 1. Introduction

The pollution of water resources by various organic pollutants poses a significant environmental concern, given their elevated toxicity even at low concentrations. Organic dyes merit special attention due to their high toxicity and their presence in wastewater from a diverse range of industrial sectors, including textiles, paper manufacturing, plastics, leather production, ink formulation, cosmetics, and food processing [1,2,3].

Various techniques have been developed for the removal of organic dyes from wastewater. Among the conventional physical methods, typical approaches include adsorption, ultrafiltration, reverse osmosis, and so forth [4]. Physical treatment methods are not environmentally sustainable, as they do not break down organic dyes; instead, they simply relocate them from one phase to another. Moreover, organic dyes, with their stability and intricate aromatic structures, are hardly destroyed by biological degradation, leading to their perseverance in the environment [5]. Chemical methods such as ozonation, chlorination, and oxidation acquire high costs, demand significant amounts of hazardous chemicals, and can lead to incomplete oxidation of dyes. Additionally, these methods produce large sludge volumes that require additional treatment and disposal.

Advancements in chemical treatment methods have enhanced oxidative degradation processes of organic pollutants (such as dyes), commonly referred to as “Advanced oxidation processes (AOPs)” [6]. These methods employ a photocatalyst to expedite the degradation of the organic pollutants without being consumed in the process. Photocatalysts function by initiating redox reactions, generating reactive species like hydroxyl and superoxide radicals, which effectively oxidize a wide array of organic pollutants [7,8]. AOPs offer numerous advantages over alternative methods, including fast and complete degradation of organic pollutants, absence of sludge formation necessitating disposal, minimal generation of harmful by-products, oxidation of pollutants to levels as low as parts per billion (ppb), and avoidance of hazardous chemical usage [7,9]. Consequently, AOPs have garnered growing interest as an efficient alternative for wastewater treatment applications.

Different types of photocatalysts are employed in water purification. In the past decade, researchers have notably focused on using metal oxide semiconductor nanoparticles as photocatalysts. These nanoparticles possess an electronic structure characterized by an empty conduction band (CB) and filled valence band (VB). When the metal oxide semiconductor absorbs photon energy exceeding its band gap energy (EBG), electrons transition from the VB to the CB, resulting in the formation of electron–hole pairs (h^+^_vb_/e^−^_cb_). The positive holes (h^+^_vb_) are a strong oxidizing agent for hydroxyl groups and water molecules, whereas electrons (e^−^_cb_) are a good reducing agent for oxygen molecules [10,11,12,13]. This redox reaction produces reactive radicals (OH^•^, O_2_^•−^) that are essential for the photodegradation of dyes into harmless components like CO_2_, H_2_O, and mineral ions, as presented in Equations (1)–(6) below [14]:

TiO_2_ + hν → TiO_2_ (e^−^_cb_ + h^+^_vb_) (1)

TiO_2_ (h^+^_vb_) + H_2_O → TiO_2_ + H^+^+ OH^•^ (2)

TiO_2_(h^+^_vb_) + OH^−^ → TiO_2_ + OH^•^ (3)

TiO_2_(e^−^_cb_) + O_2_ → TiO_2_ + O_2_^•−^ (4)

O_2_^•−^ + H^+^ → HO_2_^•^ (5)

Organic pollutants + OH^•^/O_2_^•−^ → degradation products (CO_2_, H_2_O, mineral ions) (6)

Among the various metal oxide nanoparticles employed in photocatalysis processes, titanium dioxide nanoparticles (TiO_2_ NPs) have received particular interest as a promising photocatalyst for water treatment endeavors. TiO_2_ NPs are chemically and biologically inert, along with notable stability against photo- and chemical corrosion. They are cost-effective and demonstrate high photoactivity [7,15]. Nevertheless, there are some limitations to TiO_2_ as a high-efficiency photocatalyst, notably the swift recombination of electron–hole pairs (e^−^_cb_/h^+^_vb_) and its inefficient absorption of visible light, attributed to its large band gap of 3.3 ev [16,17]. Generally, enhancing TiO_2_’s performance and elevating its photocatalytic activity can be achieved by lowering the band gap energy to enhance absorption of visible light and increasing the charge separation lifetime by improving electron transfer from photoexcited TiO_2_ to electron acceptors [18,19]. To overcome these limitations, TiO_2_ nanoparticles can be combined with other functional materials to form nanocomposites with improved photocatalytic performance [16,18,20].

Graphene consists of two-dimensional carbon atoms, with each carbon atom being bonded to another through sp^2^-hybridization bonds, forming thick planner sheets with hexagonal structures [21,22]. Its remarkable properties, such as high conductivity, high mobility, high specific surface area, and cost-effectiveness [23], make graphene a valuable material for various applications, including sensors, energy storage, solar cells, drug delivery, and catalysis [24,25]. Graphene nanosheets have a propensity to aggregate due to robust π-π stacking and van der Waal interactions, which limits their dispersibility and conductivity, thereby constraining their utility across various domains [26,27]. To prevent this agglomeration, graphene can be functionalized with other small molecules or polymers, enhancing its dispersal, conductivity, and surface area [28]. Many studies have demonstrated the successful utilization of graphene sheets and functionalized graphene to modify the photoactivity of TiO_2_ NPs. This is achieved through the efficient transportation of electrons via a large π-conjugation system and 2-D planner structure, thereby minimizing electron–hole recombination and narrowing the band gap energy of TiO_2_ NPs [29].

Cyclodextrins (CDs) are cyclic oligosaccharides consisting of six to eight D-glucopyranose units connected by 1,4-α-glycosidic bonds, resulting in a hydrophilic outer surface and a hydrophobic inner surface. There are three known types of CDs, α-, β -, and γ-CD, composed of six, seven, or eight glucopyranose units, respectively [30,31,32]. Recently, Jayanayak et al. [33] reported that water-insoluble polymers based on cyclodextrin might be employed as a cheaper alternative to expensive adsorbents for the removal of malachite green (MG) dye from aqueous solutions. Many studies reported the significance of CDs in enhancing the photocatalytic activity of TiO_2_ NPs. Einafshar et al. [34] reported that the inherent properties of β-cyclodextrin create a stable aqueous graphene solution that is capable of self-assembling in situ-grown TiO_2_ nanoparticles on graphene nanosheets. Therefore, the incorporation of β-CD molecules onto the TiO_2_ photocatalyst surface boosts its stability against aggregation and enhances its photocatalytic activity. CDs function by forming a “channel” or “bridge” between the photocatalyst surface and dye molecules, facilitating the delivery of many dye molecules through its hydrophobic cavity and improving charge transfer from the photoexcited photocatalyst to the electron acceptors. This process generates more radicals and inhibits the recombination of electron–hole pairs (e^−^_cb_/h^+^_vb_) [35,36]. The role of β-cyclodextrin (β-CD) is supported by its ability to form inclusion complexes with organic molecules, thereby enhancing their adsorption near active photocatalytic sites, as well as by the presence of functional groups that promote effective interaction between rGO sheets and TiO_2_ nanoparticles, leading to improved dispersion and reduced agglomeration.

Noble metals such as Ag and Pt are widely used to enhance the photocatalytic performance of metal oxide nanoparticles due to their ability to induce localized surface plasmon resonance (LSPR), which arises from the collective oscillation of free electrons under light irradiation [37]. The deposition of Ag or Pt nanoparticles on the surface of TiO_2_ nanoparticles can significantly improve photocatalytic efficiency by facilitating the generation of reactive oxygen species and suppressing the recombination of photogenerated electron–hole pairs. In addition, noble metals can serve as electron acceptors, promoting charge separation and transfer, which may contribute to a reduction in the effective band gap energy of the photocatalytic system [38,39]. Although noble metals increase the material cost, small loading amounts (e.g., 5 wt.%) can substantially enhance catalytic efficiency, making them feasible for advanced photocatalytic systems. Additionally, Ag is significantly less expensive than Pt, which may make Ag-based catalysts more attractive for large-scale wastewater treatment applications. Although noble metals significantly enhance photocatalytic activity, their economic cost must be considered when evaluating large-scale applications. Platinum is known for its excellent catalytic performance but is relatively expensive compared with other metals. In contrast, silver is considerably more affordable while still providing strong plasmonic enhancement and improved charge separation. In the present study, the Ag-decorated nanocomposite achieved a degradation efficiency of approximately 97%, which is very close to that obtained with Pt-based catalysts. Considering the small difference in performance and the substantially lower cost of Ag, Ag-modified photocatalysts may represent a more economically viable option for practical wastewater treatment applications.

Despite the extensive research on TiO_2_-based photocatalysts, challenges such as rapid electron–hole recombination and limited adsorption capacity still restrict their photocatalytic efficiency in wastewater treatment applications. While previous studies have explored the modification of TiO_2_ with graphene derivatives, cyclodextrins, or noble metals individually, the synergistic integration of β-cyclodextrin-functionalized reduced graphene oxide with noble-metal-decorated TiO_2_ nanoparticles has not been sufficiently investigated.

This article presents the synthesis of a nanocomposite based on TiO2 NPs and β-CD@rGO, which is assessed as a photocatalyst through the investigation of MB degradation under simulated solar radiation. Moreover, the nanocomposite is doped with Ag and Pt nanoparticles, and the impact of doping on both the photocatalytic activity and the band gap of TiO2 is examined and reported. In this study, a novel β-cyclodextrin-functionalized rGO–TiO_2_ nanocomposite decorated with Ag and Pt nanoparticles was successfully synthesized and evaluated for photocatalytic degradation of methylene blue under light irradiation. The incorporation of β-cyclodextrin is expected to enhance pollutant adsorption through host–guest interactions, while rGO facilitates efficient electron transport and noble metals introduce localized surface plasmon resonance (LSPR) effects that improve light absorption and charge separation. The combined effect of these components is expected to significantly enhance photocatalytic performance and stability. Therefore, this work aims to investigate the synergistic role of β-CD, rGO, and noble metals in improving the photocatalytic degradation efficiency of TiO_2_-based nanocomposites for wastewater treatment applications.

## 2. Experimental Procedure

### 2.1. Materials

Titanium (IV) oxide, anatase nanopowder (99.7%), methylene blue (MB) powder, and hydrazine hydrate NH_2_-NH_2_·H_2_O (80%) were purchased from Sigma-Aldrich (St. Louis, MO, USA). Graphite powder and Beta-cyclodextrin hydrate (99%) were purchased from Acros Organic (Geel, Belgium). Potassium permanganate (purity ≥ 99.5%) (KMnO_4_) was obtained from Park Scientific (Northampton, UK). Sodium hydroxide (NaOH) (purity ≥ 99.5%) was purchased from BBC Chemicals (Lancashire, UK), and Hydrogen peroxide (H_2_O_2_) (purity 30%) was purchased from AppliChem (Darmstadt, Germany). Sulfuric acid (H_2_SO_4_) (purity 95–98%) was purchased from Schalru (Tokyo, Japan), and silver nitrate (AgNO_3_) was purchased from Xilong Chemical Co., Ltd. (Shantou, China). Potassium tetra chloroplatinate (II) (K_2_PtCl_4_) was purchased from Oakwood Chemicals (Estill, SC, USA).

### 2.2. Preparation of Graphene Oxide (GO)

Graphene oxide was prepared via the modified Hummers method with minor modifications [40]. Following this method, 2 g of graphite was added to 47 mL of concentrated H_2_SO_4_ and vigorously stirred in a conical flask. The flask was then placed in an ice bath and cooled to 0 °C for 30 min. Subsequently, 7 g of (KMnO_4_) was gradually added while keeping the temperature at 20 °C in a water bath. After the KMnO_4_ addition, the mixture was transferred to a water bath at 35 °C and stirred for 2 h.

The resultant dark green solution underwent dilution with 100 mL of deionized water, followed by the addition of 10 mL of 30% (H_2_O_2_). The mixture underwent centrifugation and was rinsed with 10% HCl and subsequently twice with deionized (DI) water. The resulting product was then dried in an oven at 105 °C for 48 h and stored for subsequent utilization.

### 2.3. Preparation of β-Cyclodextrin-Functionalized Graphene (β-CD@rGO)

The β-CD@rGO was synthesized as follows: First, 20 mL homogeneous graphene oxide (0.5 mg/mL) was mixed with 20 mL of β-CD aqueous solution (80 mg) and 300 μL of ammonia solution, followed by the addition of 20 μL of hydrazine hydrate solution (80%). After vigorous stirring for several minutes, the vial was immersed in a water bath at 60 °C for 3.5 h, yielding a stable black dispersion. The product was then isolated via centrifugation and washed twice with deionized water to remove free β-CD molecules [41].

### 2.4. Preparation of TiO_2_/β-CD@rGO Nanocomposites

The utilized procedure employed in this study was derived from the work of Mohamed et al. [42], with minor adjustments. Initially, different amounts of β-CD@rGO (50 mg, 100 mg, and 250 mg) were individually dispersed in 100 mL DI water by sonication for 1 h to achieve a uniform suspension. Subsequently, 500 mg TiO_2_ NPs was dispersed in 50 mL DI water by sonication for 40 min and then gradually added to the β-CD@rGO dispersion under sonication. Once the addition was completed, the mixture underwent further sonication for another 30 min to ensure adequate contact between TiO_2_ NPs and β-CD@rGO. Following this, the TiO_2_/β-CD@rGO nanocomposite was separated by centrifugation at 600 rpm for 15. The obtained gray product was washed multiple times with DI and subsequently dried overnight at 60 °C.

Three distinct samples were prepared, each with varying weight percentages of β-CD@rGO (10, 20, and 50 wt.%), denoted as sample 1, sample 2, and sample 3, respectively.

### 2.5. Preparation of β-CD@rGO/TiO_2_ with Silver and Platinum Nanoparticles

Doping of β-CD@rGO/TiO_2_ with silver and platinum nanoparticles was accomplished through the following procedure: Firstly, 150 mg of the prepared sample 3 was evenly dispersed into two beakers, each containing 100 mL deionized water, by ultrasonication for 30 min. Subsequently, 20 mL of silver nitrite solution (11.8 mg) in DI was gradually added to the first beaker, while a 20 mL K_2_PtCl_4_ solution (15.9 mg) in DI was gradually added to the second beaker, both under sonication for 10 min. Ag and Pt nanoparticles were obtained by the reduction of Ag^+^ and PtCl_4_^2−^ ions using hydrazine. Subsequently, 1 mL of hydrazine was added dropwise to each resulting dispersion over 30 min under magnetic stirring. Following this, each product was centrifuged and washed multiple times with DI water to remove excess hydrazine.

### 2.6. Characterization

The synthesized nanocomposites were characterized using Fourier Transform Infrared (FT-IR) spectroscopy with Tensor II, a benchtop Bruker FTIR instrument (Bruker, Ettlingen, Germany). Spectra were recorded across the range of 400–4000 cm^−1^.

The X-ray diffraction (XRD) pattern was obtained using a computer-controlled Hiltonbrooks XRD diffractometer (Hiltonbrooks Ltd., Crewe, UK) equipped with a Philips PW 1050 diffractometer (Amsterdam, The Netherlands), utilizing Cu-K radiation (λ = 1.54056 Å). The scan covered a range of 2θ from 0 to 80°, with a step size of 0.02°. Scanning electron microscope images were taken using QUANTA450 FEG Scanning Electron Microscope (FEI, Hillsboro, OR, USA).

The photocatalytic activity of the synthesized samples in degrading MB dye was evaluated at room temperature under solar illumination utilizing the PECCEL PEC-L01 Portable Solar Simulator equipped with a 150 W Short-arc Xe lamp (Peccell Technologies, Inc., Kanagawa, Japan). The absorption spectra of the MB dye solutions were monitored using UV-VIS spectrophotometer (Shimadzu, Kyoto, Japan, Double Beam UV-2500PC) with a quartz cell, covering a range of 400–800 nm.

### 2.7. Photocatalytic Activity Experiment

**Figure 1:**
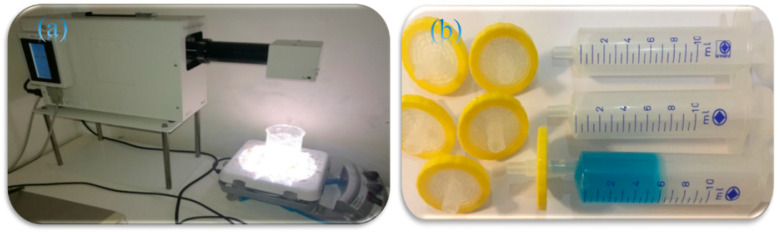
(**a**): Photodegradation of MB dye under illumination of sunlight using PECCEL PEC-l01 portable solar simulator. (**b**): Syringe and syringe filter assembly used for sampling.

The photocatalytic activity of the prepared samples was investigated through the degradation of methylene blue (MB) dye, serving as a model for organic pollutants, under simulated solar illumination. In this experiment, 50 mg of the prepared photocatalyst was dispersed in 200 mL of MB dye aqueous solution. The dye solution was prepared by dissolving 10 mg of MB powder in 1 L DI water, resulting in a concentration of 10 mg/L (3.13 × 10^−5^ M). The photocatalyst was dispersed into the MB solution by sonication for 10 min, followed by stirring for 30 min in a dark environment to achieve adsorption/desorption equilibrium. Subsequently, the prepared solution was exposed to the solar simulator, as shown in Figure 1, for a duration of 90 min. Throughout the experiment, 5 mL samples were collected at 10 min intervals. Each sample was then filtered and analyzed using a UV-VIS spectrophotometer [43].

Each of the prepared nanocomposites was individually tested to determine which nanocomposite exhibited the optimum efficiency in the photodegradation of MB under the same experimental conditions.

### 2.8. Recycling Experiment

The recyclability of the nanocomposite showing the best performance was examined through 3 cycles of repeated usage. Following each cycle, the nanocomposite was separated via centrifugation, washed multiple times with DI water to remove adsorbed MB, and subsequently dried in an oven at 50 °C. The dried nanocomposite was then reused for the degradation of MB under the same experimental conditions outlined in Section 2.7.

During the recycling procedure, the catalyst was washed multiple times with deionized water after each photocatalytic cycle to remove any residual or adsorbed MB molecules from the catalyst surface. This washing step allows for desorption of MB from the nanocomposite surface before reusing. Additionally, during photocatalysis, most of the MB molecules that are adsorbed on the catalyst surface are degraded through oxidative reactions induced by reactive oxygen species.

### 2.9. Analysis of Band Gap

To determine the band gap energy (E_BG_) of pure TiO_2_ NPs and TiO_2_ NPs with various modifiers, each nanocomposite was dispersed in 100 mL of deionized water. Subsequently, a 5 mL sample was extracted, and their light absorption was measured at room temperature. The analysis was carried out in the wavelength range from 300 to 800 nm using a UV-VIS spectrophotometer [44]. The collected data was utilized to calculate the optical band gap using Tauc’s equation.

## 3. Results and Discussion

### 3.1. FT-IR Analysis

**Figure 2:**
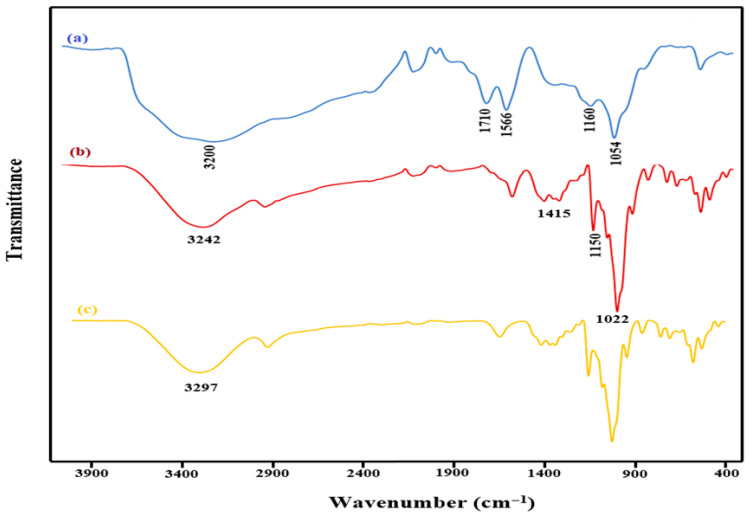
FT-IR spectra of (**a**) GO, (**b**) β-CD@rGO, and (**c**) β-CD.

Figure 2 shows the FT-IR spectra for (a) GO, (b) β-CD, and (c) β-CD@rGO. In the GO spectrum, the presence of oxygen-containing functional groups is evident, with strong absorption peaks being observed at 1710 cm^−1^, 1566 cm^−1^, and 3500 cm^−1^, which is attributed to the carboxyl group, (C=O) stretching of COOH groups, alkene (C=C) stretching vibrations, and O-H stretching, respectively. Additionally, a minor peak at 1054 cm^−1^ corresponds to epoxide group (C-O-C) vibrations, confirming the formation of GO [45].

β-CD becomes covalently attached to the GO surface through a reaction of -OH groups that are present in β-CD, with the oxygen functional groups being present on the GO surface. The peak observed at 1150 cm^−1^ corresponds to the C-O-C (glycosidic linkage) stretching/O-H bending vibrations, while the peak at 1415 cm^−1^ is attributed to the -CH/O-H bending vibrations. Additionally, peaks at 1022 and 1075 cm^−1^ are a result of coupled C-O/C-C stretching. Remarkably, the peak at 3297 cm^−1^ representing -OH in β-CD shifts to 3242.59 cm^−1^, indicating successful attachment of β-CD molecules to rGO sheets [46].

**Figure 3:**
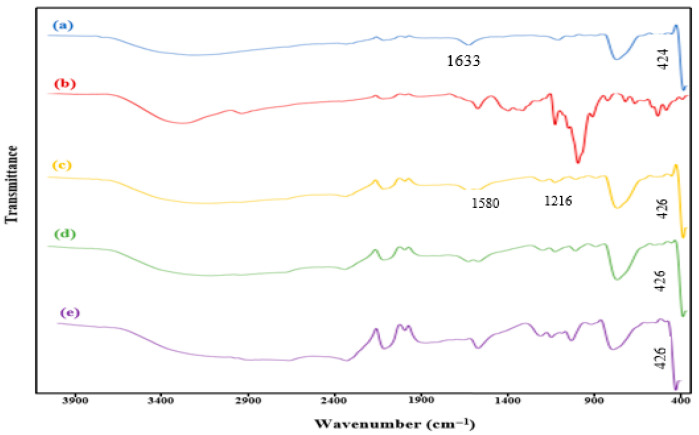
FT-IR spectra of (**a**) TiO_2_ NPs, (**b**) β-CD@rGO, (**c**) sample 1, (**d**) sample 2, (**e**) sample 3.

Figure 3 shows the FT-IR spectra for (a) TiO_2_, (b) β-CD@rGO, and (c, d, e) β-CD@rGO/TiO_2_ (sample 1, sample 2, and sample 3, respectively). TiO_2_ nanoparticles exhibit broad peaks ranging from 2500 to 3700 cm^−1^, indicative of the O-H group of water, suggesting the existence of free hydroxyl groups. Furthermore, the wide and broad peaks ranging from 570 to 900 cm^−1^ are attributed to the stretching vibration of Ti-O-Ti and Ti-O-C bonds. The peak observed at 1633 cm^−1^ in pure TiO_2_ is attributed to the in-plane bending vibration of surface -OH groups [38].

Upon comparing the spectra of pure TiO_2_ (Figure 3a) with that of the β-CD@rGO/TiO_2_ nanocomposite (Figure 3c–e), it becomes evident that the peak at 1633 cm^−1^ undergoes a decrease and a shift to 1580.75 cm^−1^ following the addition of β-CD@rGO/TiO_2_. Additionally, the Ti-O stretching vibration peak that is associated with the low frequency experiences a slight shift to 426.82 cm^−1^ compared to pure TiO_2_ (centered at 424.62 cm^−1^) [47].

Moreover, Figure 3c–e show a new stretching vibration of the C-O-C bond at 1216 cm^−1^, attributed to the glucose linkages in β-CD structure. This observation serves to confirm the presence of β-CD molecules within the synthesized nanocomposites.

**Figure 4:**
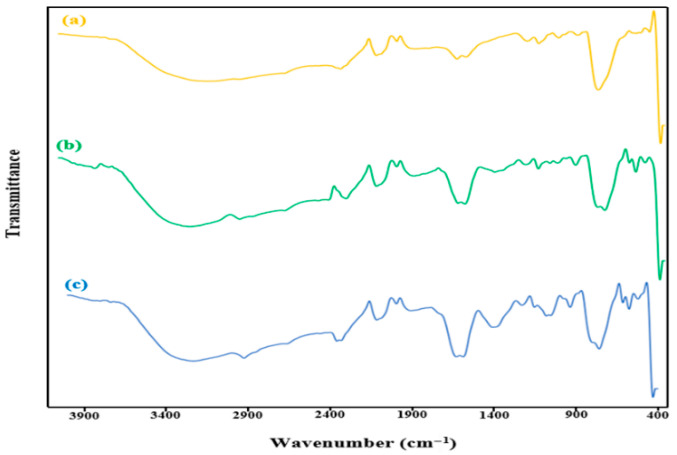
FT-IR spectra of (**a**) β-CD@rGO/TiO_2_, (**b**) β-CD@rGO/TiO_2_/Ag, (**c**) β-CD@rGO/TiO_2_/Pt.

Figure 4 shows the FTIR spectra of the metal-loaded nanocomposite. A notable observation is the shift in the glycoside peak (C-O-C) from 1216 cm^−1^ to 1226.26 cm^−1^ and the stretching vibration peak of Ti-O towards a higher wavenumber. Additionally, the peak associated with (Ti-O-Ti/Ti-O-C) shifts from 796.96 cm^−1^ to a lower wavenumber at 756.18 cm^−1^. These alterations in the FT-IR spectrum suggest the attachment of silver and platinum to β-CD@rGO/TiO_2_. The appearance of a new peak within the range of 1390–1411 cm^−1^ may be attributed to the presence of Ag and Pt metals. The appearance of a band in the range of 1390–1411 cm^−1^ may be associated with surface structural modifications and metal–oxygen interactions resulting from the incorporation of Ag and Pt nanoparticles onto the TiO_2_ framework, as reported in previous studies [48].

Comparing the FT-IR spectrum of the nanocomposite with Ag to that with Pt, they exhibit similarities, despite some distinctions. In the case of Pt, the peaks at 1405 cm^−1^ and 1078 cm^−1^ are observed with less intensity and are shifted to lower wavenumbers, which can be attributed to the higher atomic weight of Pt compared to Ag.

### 3.2. XRD Analysis

**Figure 5:**
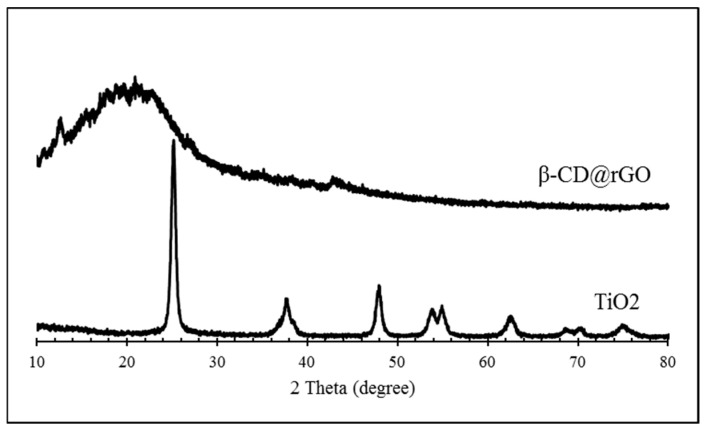
The XRD patterns of β-CD@rGO, and TiO_2_ NPs.

The crystallographic structures of the prepared samples were analyzed using X-ray diffraction. Figure 5 shows the XRD patterns of β-CD@rGO and TiO_2_ nanoparticles. The functionalized graphene exhibited a broad diffraction peak at 2θ = 20°, attributed to interlayer spacing resulting from either the presence of CD molecules or the removal of functional oxygen groups from GO and its complete reduction to rGO. Additionally, a weak peak at 44° was observed. These two peaks can be associated with the (002) and (101) diffraction for typical graphite carbon, indicating the presence of rGO [49].

**Figure 6:**
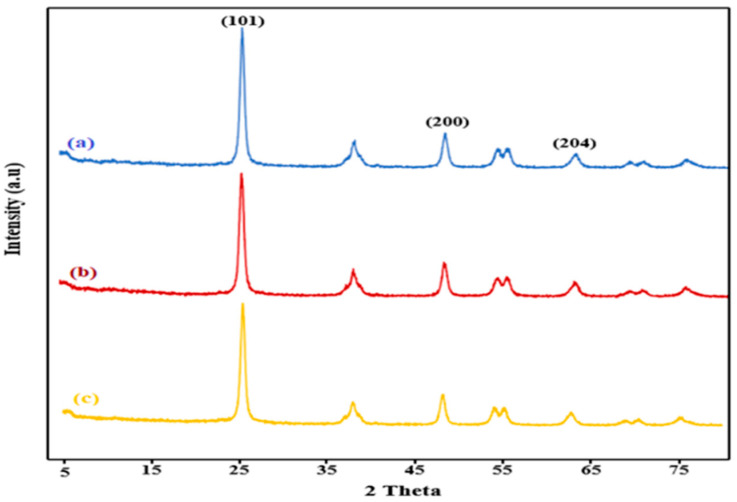
The XRD patterns of (**a**) 10% of β-CD@rGO/TiO_2_ sample (1), (**b**) 20% of β-CD@rGO/TiO_2_ sample (2), and (**c**) 50% of β-CD@rGO/TiO_2_ sample (3).

Figure 6 illustrates the XRD pattern of sample 1, sample 2, and sample 3. The pattern displays all the characteristic peaks of anatase TiO_2_. Peaks located at 25°, 37.8°, 47.8°, 54.3° and 62.71° can be attributed to the (101), (004), (200), (105), and (204) crystal planes of tetragonal anatase TiO_2_ [50]. However, the primary peaks that are typically observed for graphene are not detected. This absence is attributed to the low quantity and weak intensity of graphene compared to the highly crystalline TiO_2_ [42]. Moreover, the figure shows the higher crystallinity of the TiO_2_ in the β-CD@rGO/TiO_2_ compared to TiO_2_ NPs, which is evident from the peak intensity and sharpness. This enhancement may originate from the improved dispersion of TiO_2_ NPs on the surface of β-CD@rGO [51].

**Figure 7:**
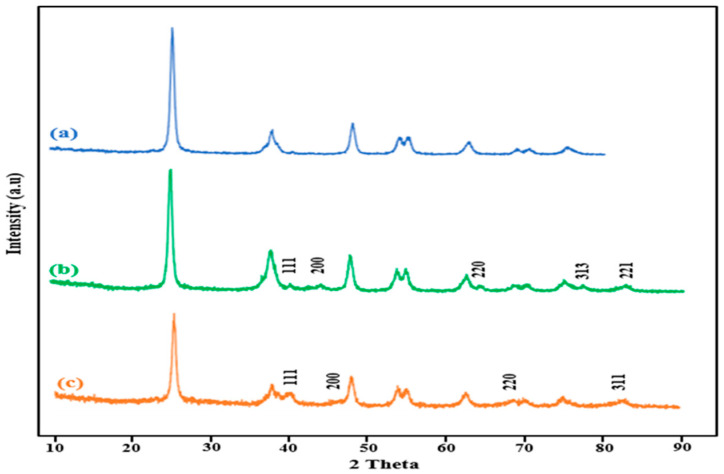
The XRD patterns of (**a**) 50% of β-CD@rGO/TiO_2_ sample 3, (**b**) β-CD@rGO/TiO_2_/Ag, (**c**) β-CD@rGO/TiO_2_/Pt.

The XRD pattern of the β-CD@rGO/TiO_2_ nanocomposite post-loading with Ag and Pt nanoparticles is shown in Figure 7. In the XRD pattern of β-CD@rGO/TiO_2_/Ag, new diffraction peaks are located at 40°, 44.28°, 64.43°, 77.47° and 82.54°, corresponding to the crystalline phases (111), (200), (220), (311) and (222) of Ag NPs, respectively [52,53]. Similarly, in the XRD pattern of β-CD@rGO/TiO_2_/Pt, new diffraction peaks are located at 39.8°, 46°, 68.1° and 81.2°, corresponding to the crystalline phases (111), (200), (220) and (311) of platinum nanoparticles, respectively [54,55]. This observation validates the successful formation of Ag and Pt nanoparticles on the surface of the synthesized nanocomposite material.

### 3.3. Morphology Analysis

**Figure 8:**
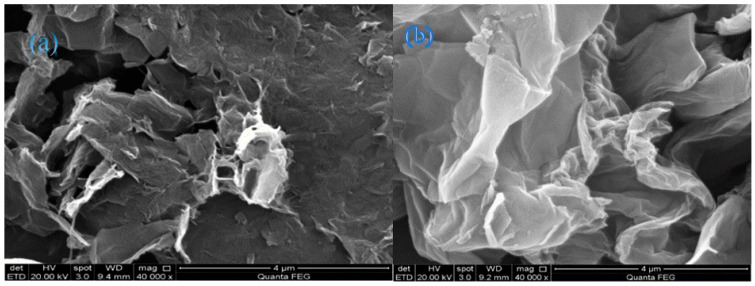
SEM images of (**a**) GO, and (**b**) β-CD@rGO.

The morphology of the synthesized nanocomposites at various stages of preparation was examined by a scanning electron microscope (SEM). Figure 8 shows SEM images of (a) GO, and (b) β-CD@rGO. The GO sheets exhibit a typical wrinkled, fabric-like structure with a high level of agglomeration. In contrast, β-CD functionalized rGO sheets display a smoother structure (flatter surface) with fewer wrinkles and minimal agglomeration. The attachment of hydrophilic β-CD across the basal plane of rGO prevents the restacking of rGO sheets, thereby reducing agglomeration [56]. This behavior is highly beneficial, as it increases the accessible surface area of the photocatalyst, thereby enhancing its efficiency.

**Figure 9:**
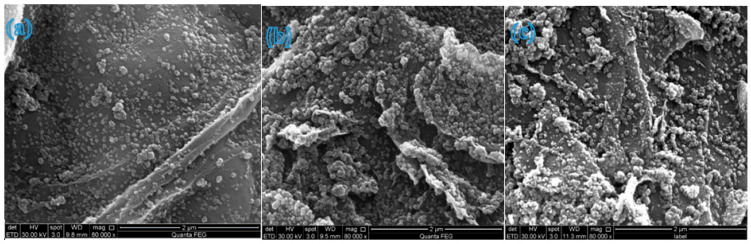
SEM images of (**a**) sample 1, (**b**) sample 2, and (**c**) sample 3.

The SEM images in Figure 9a–c represent samples 1, 2, and 3, respectively. These images illustrate a homogeneous and even distribution of TiO_2_ NPs across β-CD@rGO sheets, which is particularly evident at lower concentrations. Moreover, the TiO2 NPs appear to be firmly attached to the rGO sheets’ surfaces. This surface structure is beneficial for enhancing adsorption and providing more active sites for photocatalytic degradation reactions [42,49]. With increasing TiO_2_ NP concentrations, a greater degree of agglomeration is observed, which is typical for NPs of this size range.

**Figure 10:**
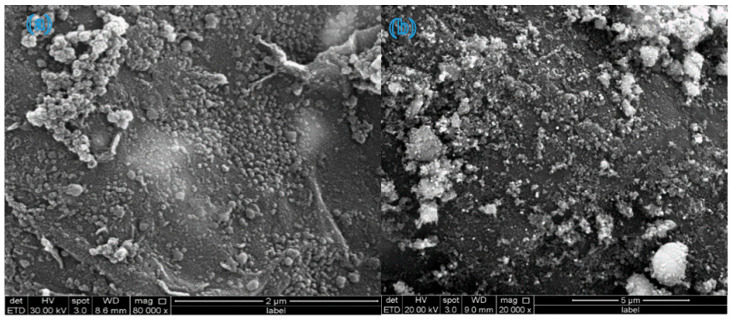
SEM images of (**a**) β-CD@rGO/TiO_2_/Ag and (**b**) β-CD@rGO/TiO_2_/Pt.

**Figure 11:**
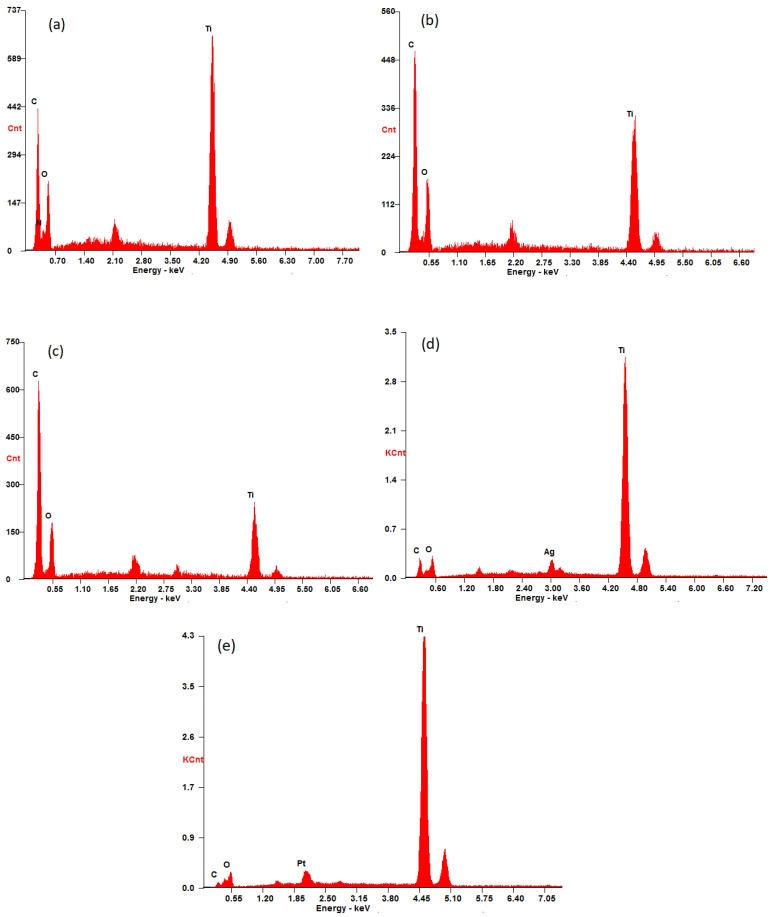
EDAX spectra of (**a**) sample 1, (**b**) sample 2, (**c**) sample 3, (**d**) Ag, and (**e**) Pt nanocomposites.

The SEM images of β-CD@rGO/TiO_2_/Ag and β-CD@rGO/TiO_2_/Pt shown in Figure 10 exhibit a similar morphology as the β-CD@rGO/TiO_2_ nanocomposite. A uniform distribution of the NPs on the surface of rGO sheets is evident. The presence of the Ag and Pt nanoparticles was confirmed using EDX analysis. The elements that are present in the prepared samples were observed by energy-dispersive X-ray spectroscopy (EDX). Figure 11a–e show the EDX spectrum of samples 1, 2, 3, Ag, and Pt, respectively. Peaks corresponding to titanium, carbon, and oxygen atoms are observed in all spectra, indicating the presence of β-CD, rGO, and TiO_2_ in all prepared samples.

In Figure 11c, the peak corresponding to carbon atoms shows higher intensity compared to Figure 11a,b, possibly due to the higher carbon content in sample 3 (50 wt.%). Following the loading of silver and platinum atoms onto β-CD@rGO/TiO2 nanocomposites, new peaks appear in the EDX spectrum at 3.00 and 2.08 kev corresponding to silver and platinum atoms, respectively. This confirms the presence of silver and platinum atoms and indicates successful loading of Ag and Pt NPs using the method of in situ chemical precipitation reduction of metal salts. The target loading was 5 wt.%, and EDX analysis confirmed the successful incorporation of Ag and Pt, although it is semi-quantitative.

### 3.4. Photocatalytic Activity

**Figure 12:**
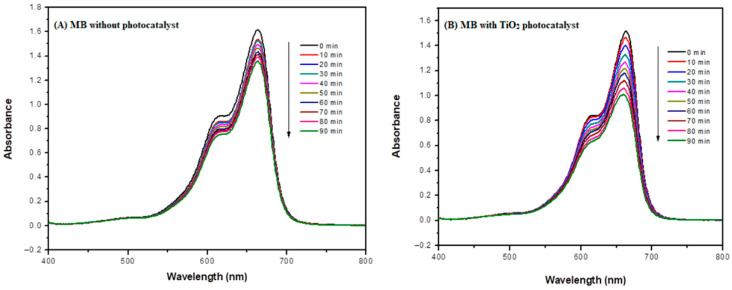

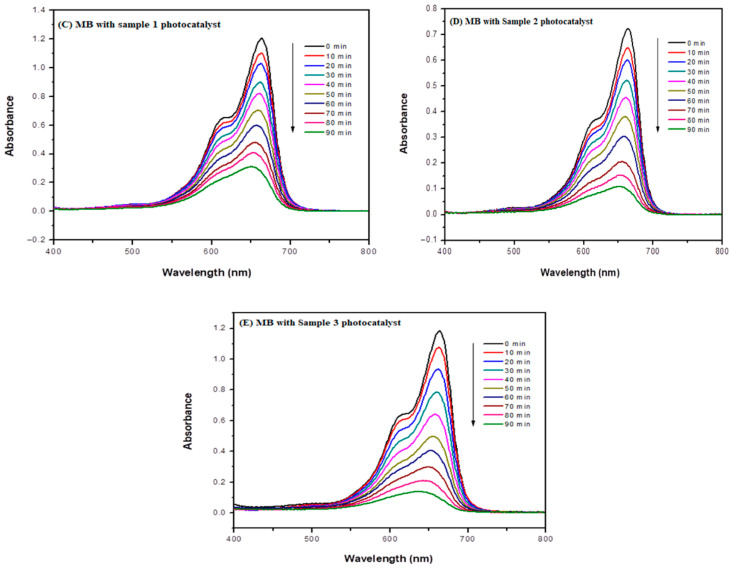
UV-VIS Spectra of MB dye with (**A**) no photocatalyst, (**B**) TiO_2_, (**C**) sample 1, (**D**) sample 2, (**E**) sample 3.

As mentioned earlier, the photocatalytic activity of the synthesized nanocomposites was evaluated by photodegradation of MB dye, serving as a model of organic pollutants under solar illumination using the PECCEL PEC-L01 solar simulator. The photocatalytic activity was evaluated for each material after every synthesis step under the same experimental conditions to study the effect of various functionalization steps on the photodegradation efficiency. The results are shown in Figure 12A–E, illustrating changes in absorbance during the photodegradation of MB at different time intervals. Measurements were conducted using a UV-VIS spectrophotometer at the maximum absorbance wavelength (λ_max_) of 664 nm for MB dye.

The degradation percentage of MB dye in an aqueous medium was calculated by using Equation (7) [8]:

(7) $$Degradation\;Rate\;\left(\%\right)=\frac{\left(A_{0}-A\right)}{A_{0}}\times 100\%\;\;$$

where *A*_0_ is the initial dye absorbance, and *A* is the dye absorbance after irradiation.

In the absence of a photocatalyst, a slight decrease in absorbance of MB dye at λ_max_ = 664 nm was observed, with only 16% photodegradation of MB being achieved within 90 min of irradiation. However, in the presence of TiO_2_ nanoparticles, accelerated photodegradation of MB dye occurred, resulting in a photodegradation yield of 35% for the same irradiation time. Functionalization of TiO_2_ with β-CD@rGO significantly enhanced its photocatalytic activity, as evidenced by the UV-VIS spectra of MB dye in Figure 12C–E. Increasing the weight percentage (wt.%) of β-CD@rGO in the nanocomposite led to a higher degradation rate. The results show that the photodegradation percentage of sample 1, sample 2, and sample 3 are 77%, 87%, and 92%, respectively.

**Figure 13:**
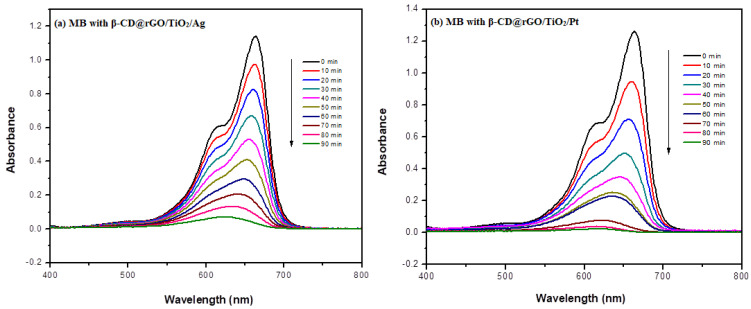
UV-VIS Spectra of MB dye with (**a**) β-CD@rGO/TiO_2_/Ag and (**b**) β-CD@rGO/TiO_2_/Pt nanocomposites.

A further enhancement in the photodegradation activity of the synthesized nanocomposite was observed in the case of Ag and Pt doping. Figure 13 shows the UV-VIS spectra of MB dye with (a) β-CD@rGO/TiO_2_/Ag and (b) β-CD@rGO/TiO_2_/Pt nanocomposites. The degradation percentage reached 99.9 and 97% for Pt and Ag doping, respectively. This result confirms the contribution of noble metals in enhancing the photocatalytic activity of semiconductor TiO_2_ nanoparticles.

**Figure 14:**
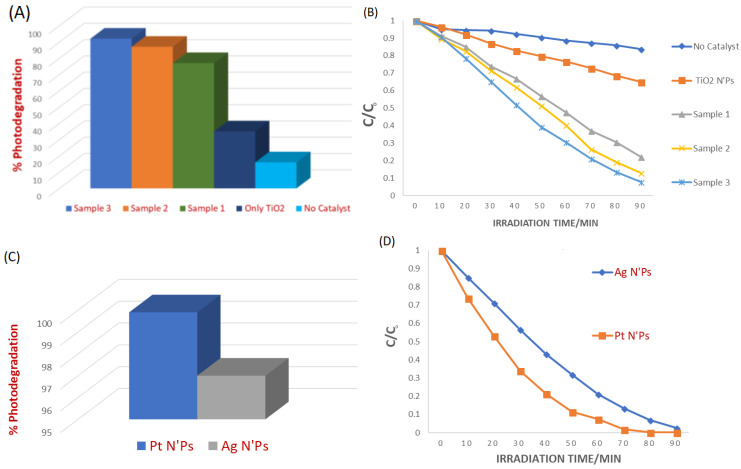
(**A**) % Photodegradation efficiency and (**B**) concentration changes in MB in the presence of all prepared nanocomposites, only TiO_2_ NPs, and without photocatalyst. (**C**) % Photodegradation efficiency and (**D**) concentration changes in MB in the presence of Pt and Ag nanocomposites. Here, C_0_ is the initial MB dye concentration, and C is the dye concentration after irradiation.

Figure 14 shows a summary of the experimental results obtained from the photodegradation of MB using the synthesized nanocomposite. Figure 14A shows the % photodegradation efficiency, while Figure 14B shows the concentration changes in MB in the presence of all prepared nanocomposites, only TiO2 NPs, and without photocatalyst among them. Figure 14 reveals that the β-CD@rGO/TiO_2_/Pt nanocomposite exhibited the highest degradation percentage and degradation rate. Both catalysts exhibit very high photocatalytic efficiency, and the difference between Pt and Ag catalysts may not be statistically significant, although Pt may offer slightly improved charge separation due to its higher work function.

### 3.5. Recycling Analysis

**Figure 15:**
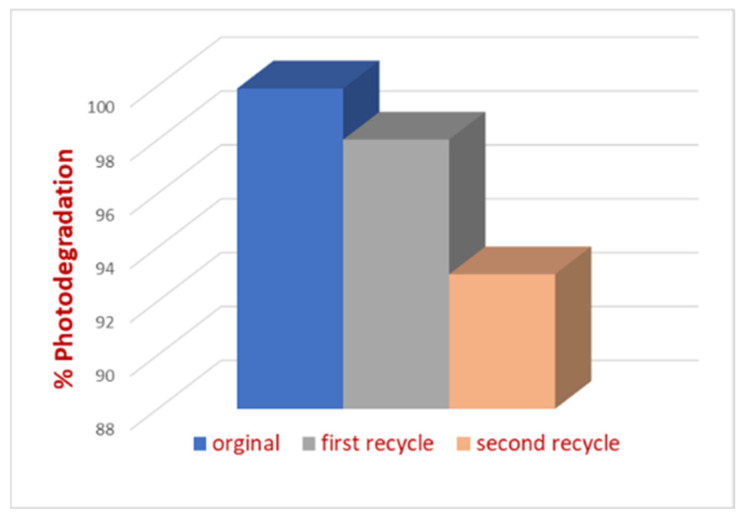
%Photodegradation of MB dye in the presence of β-CD@rGO/TiO_2_/Pt photocatalyst after 3 consecutive cycles.

Water treatment applications often require treating large amounts of water. For practical and economic reasons, the ability to recycle a photocatalyst with minimal loss of efficiency is crucial. In this study, the photocatalytic activity of the synthesized nanocomposite was evaluated over multiple usage cycles. After each cycle, the photocatalyst was washed several times with DI water to remove adsorbed MB from its surface. The analysis was performed using β-CD@rGO/TiO_2_/Pt, and the results are shown in Figure 15. It is evident that the activity of the photocatalyst decreased after each usage cycle, likely due to the attrition of NPs from the surface during repeated washing, sonication, and mixing cycles. However, the photocatalyst retained over 90% activity after three consecutive usage cycles. The electron transfer role of rGO and recyclability importance was highlighted by Donga et al. [57].

### 3.6. Band Gap Analysis

UV-VIS absorption analysis was utilized to determine the band gap of TiO_2_ NPs. Based on the absorption spectra, the optical band gap energy was determined by using Tauc’s equation [58]:

(8) $$\alpha h\nu =B{\left(h\nu -E_{BG}\right)}^{2}$$

where *α* is the optical absorption coefficient, *B* is a constant, *E_BG_* is a band gap energy, and the photon energy is given by *E* = *hν* = hc/λ= 1240/λ eV.

**Figure 16:**
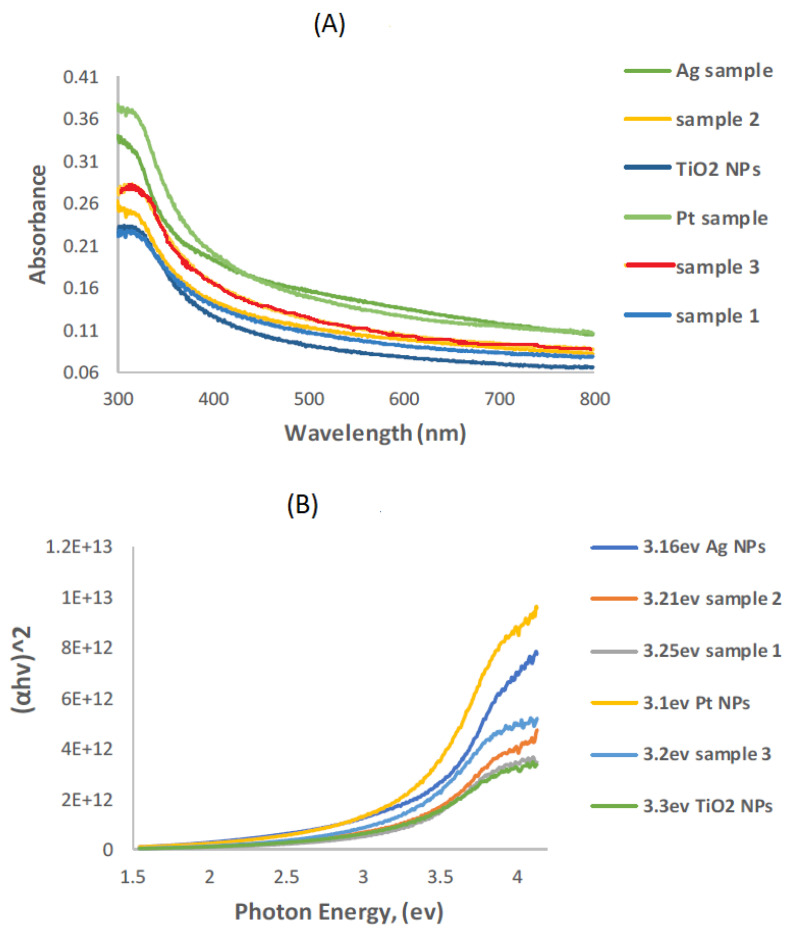
(**A**) UV-VIS absorption spectra and (**B**) plot of (α hν)^2^ vs. (hν) for TiO_2_ NPs, and all prepared nanocomposites.

Figure 16A shows the UV-VIS absorption spectra recorded for pure TiO_2_ NPs and all prepared nanocomposites. The absorption edge of TiO_2_ NPs shifts to a higher wavelength (red shift) when combined with modifier materials. The band gap energy values were obtained through a plot of (αhν)^2^ vs. E (hν), as shown in Figure 16B. From this plot, it can be observed that the band gap energy (E_BG_) values for TiO_2_ NPs after combination with β-CD@rGO, Ag, and Pt NPs are lower than those for pure TiO_2_ NPs. Additionally, the band gap energy of TiO_2_ decreases with increasing amounts of β-CD@rGO. The results are summarized in Table 1.

A comprehensive overview of recent advances in TiO_2_-based photoelectrocatalysis (PEC), with an emphasis on material design strategies to enhance visible light responsiveness and charge carrier dynamics, was presented recently by Liang et al. [59]. Key approaches—including elemental doping, defect engineering, heterojunction construction, and plasmonic enhancement—are systematically discussed in relation to their roles in modulating energy band structures and promoting charge separation. 

### 3.7. Proposed Photocatalytic Mechanism

The enhanced photocatalytic activity of the noble-metal-decorated β-CD/rGO–TiO_2_ nanocomposite can be attributed to the synergistic interaction among its components. Upon light irradiation, TiO_2_ nanoparticles absorb photons with energy equal to or greater than their band gap, generating electron–hole pairs. The photogenerated electrons are transferred from the conduction band of TiO_2_ to the rGO sheets, which act as an efficient electron transport pathway due to their high electrical conductivity. This transfer effectively suppresses electron–hole recombination [60].

The presence of noble metal nanoparticles (Ag or Pt) further enhances photocatalytic performance through the localized surface plasmon resonance (LSPR) effect, which improves visible light absorption and promotes charge separation. These metal nanoparticles can also act as electron sinks, facilitating the transfer of photogenerated electrons and prolonging charge carrier lifetimes.

Meanwhile, β-cyclodextrin molecules provide additional adsorption sites through host–guest interactions, which enhance the concentration of organic pollutant molecules near the photocatalyst surface [61]. The photogenerated holes react with water molecules or hydroxide ions to produce hydroxyl radicals (•OH), while the transferred electrons react with dissolved oxygen to generate superoxide radicals (O_2_•^−^). These highly reactive species subsequently oxidize and degrade methylene blue molecules into smaller and less harmful compounds such as CO_2_ and H_2_O. The proposed photocatalytic mechanism is based on the band structure analysis and previously reported studies. However, to provide direct experimental evidence, future investigations will incorporate radical scavenger experiments using selective quenchers (e.g., tert-butyl alcohol for •OH and p-benzoquinone for O_2_•^−^) to identify the dominant reactive species and validate the degradation pathway.

## 4. Conclusions

In this study, we presented the synthesis of a nanocomposite consisting of TiO_2_ nanoparticles functionalized with β-CD@rGO, with further decoration using Ag and Pt nanoparticles. The structure, surface morphology, and chemical composition of all prepared nanocomposites were investigated using XRD, SEM, FT-IR, and EDAX. Photodegradation of MB, as a model for organic pollutants, was measured using a UV-VIS spectrophotometer. The results indicated enhanced activity of the synthesized nanocomposite compared to pure TiO_2_ nanoparticles. Photocatalytic activity increased with the increase in wt.% of β-CD@rGO. Incorporating Ag and Pt nanoparticles further enhanced the photocatalytic activity of the nanocomposite. Loading 5 wt.% of Pt nanoparticles onto a 50 wt.% of β-CD@rGO/TiO_2_ nanocomposite achieved 99.9% degradation of MB. Recycling experiments showed the synthesized nanocomposite’s stability, photocatalytic activity, and reusability under repeated use. The nanocomposite maintained a photoactivity of over 90% after three consecutive cycles. Band gap analysis showed a decrease in the band gap of TiO_2_ NPs due to the use of different modifiers.

## Figures and Tables

**Table 1:** Band gap energy of the synthesized photocatalyst nanocomposites.

Photocatalyst	TiO_2_ NPs	10% β-CD@rGO/TiO_2_	20% β-CD@rGO/TiO_2_	30% β-CD@rGO/TiO_2_	β-CD@rGO/TiO_2_/Ag	β-CD@rGO/TiO_2_/Pt
Band gap energy (EV)	3.3	3.25	3.21	3.2	3.16	3.1

## Data Availability

The original contributions presented in this study are included in the article. Further inquiries can be directed at the corresponding authors.
